# The associations between asthma and common comorbidities: a comprehensive Mendelian randomization study

**DOI:** 10.3389/fmed.2023.1251827

**Published:** 2023-11-15

**Authors:** Xuezhao Wang, Yuchen Huang, Xiaochen Li, Yuanzhou He, Xiansheng Liu

**Affiliations:** ^1^Department of Pulmonary and Critical Care Medicine, Tongji Hospital, Tongji Medical College, Huazhong University of Science and Technology, Wuhan, China; ^2^Key Laboratory of Pulmonary Diseases of National Health Commission, Tongji Hospital, Tongji Medical College, Huazhong University of Sciences and Technology, Wuhan, China

**Keywords:** comorbidities, Mendelian randomization, asthma, genome-wide association study, allergic diseases

## Abstract

**Background:**

Asthma is a chronic respiratory disease and is often associated with multiple comorbidities. The causal relationship between asthma and these comorbidities is still unclear. This study aimed to investigate the association between genetically predicted asthma and common comorbidities.

**Methods:**

After searching PubMed and GWAS summary statistics, we identified 26 comorbidities of asthma. The causal relationship between asthma and comorbidities was assessed in two independent GWASs by bidirectional Mendelian randomization, followed by validation of the results using a multivariate Mendelian randomization analysis and several sensitivity analyses.

**Results:**

In the bidirectional Mendelian randomization analysis, chronic sinusitis [odds ratio (OR) = 1.54, *p* = 1.40 × 10^−5^], atopic dermatitis (OR = 1.36, *p* = 9.37 × 10^−21^), allergic conjunctivitis (OR = 2.07, *p* = 4.32 × 10^−6^), and allergic rhinitis (OR = 1.53, *p* = 5.20 × 10^−6^) were significantly associated with increased asthma risk. Hyperthyroidism (OR = 1.12, *p* = 0.04) had a potential increased risk for asthma. For the reverse direction, asthma showed significant associations with an increased risk of chronic obstructive pulmonary disease (OR = 1.24, *p* = 2.25 × 10^−9^), chronic sinusitis (OR = 1.61, *p* = 5.25 × 10^−21^), atopic dermatitis (OR = 2.11, *p* = 1.24 × 10^−24^), allergic conjunctivitis (OR = 1.65, *p* = 6.66 × 10^−35^), allergic rhinitis (OR = 1.90, *p* = 2.38 × 10^−57^), and a potential higher risk of allergic urticaria (OR = 1.25, *p* = 0.003).

**Conclusion:**

This study suggested a significant bidirectional association of chronic sinusitis, atopic dermatitis, allergic conjunctivitis, and allergic rhinitis with asthma. In addition, hyperthyroidism was associated with an increased risk of asthma and asthma increased the risk of chronic obstructive pulmonary disease and allergic urticaria.

## 1 Introduction

Asthma is a heterogeneous disease, usually characterized by chronic airway inflammation. It is defined by the history of respiratory symptoms, such as wheeze, shortness of breath, chest tightness and cough, that varyover time and in intensity, together with variable expiratory airflow limitation (1). Patients with asthma tend to suffer from more pulmonary and extrapulmonary comorbidities, including obstructive sleep apnea, sleep disorders, and obesity (2–4), which can affect asthma clinical symptoms and severity, increasing healthcare costs (5, 6). At the same time, allergic and non-allergic comorbidities of asthma are also of increasing interest as they may be highly relevant to the pathophysiological mechanisms of asthma (7, 8).

Recognition and appropriate management of comorbidities appear to improve asthma outcomes (9). However, it is not clear whether these conditions contribute to the development of asthma, share genetic and environmental factors with asthma, or may be biased by confounding variables and inaccurate data. On the other hand, since asthma is still a poorly understood disease, it is possible to shed light on the pathophysiological mechanisms of asthma by studying the relationship between comorbidities and asthma.

However, it is difficult to elucidate the causal relationship between asthma and these comorbidities because observational studies are often susceptible to potential residual confounding and reverse causality issues. Well-designed randomized controlled trials (RCTs) are the gold standard for causal inference, but they are costly, laborious, and have practical and ethical limitations. Better methods are therefore required to evaluate the causal relationship between asthma and comorbidities to comprehend the etiology of asthma and improve clinical outcomes for patients.

Mendelian randomization (MR) is an emerging method for reliable causal inference by using genetic variations that are robustly associated with exposures as instrumental variables (IVs) (10). MR minimizes confounding and reverse causality bias because genetic variations are randomly assigned and fixed prenatally, independent of disease onset and progression. Therefore, Mendelian randomization is a new cost-effective method to assess the reliable causal relationship between asthma and comorbidities.

Some comorbidities, including gastrointestinal disorders, atopic dermatitis, obesity, insomnia, and epilepsy, have been investigated in previous MR studies (11–15). To date, most comorbidities have not been evaluated using the MR approach. Leveraging large-scale genome-wide association studies (GWAS)‘s summary data, this study aims to explore the causal relationship between 26 comorbidities and asthma in a two-sample MR framework.

## 2 Methods

### 2.1 Study design

**Figure 1:**
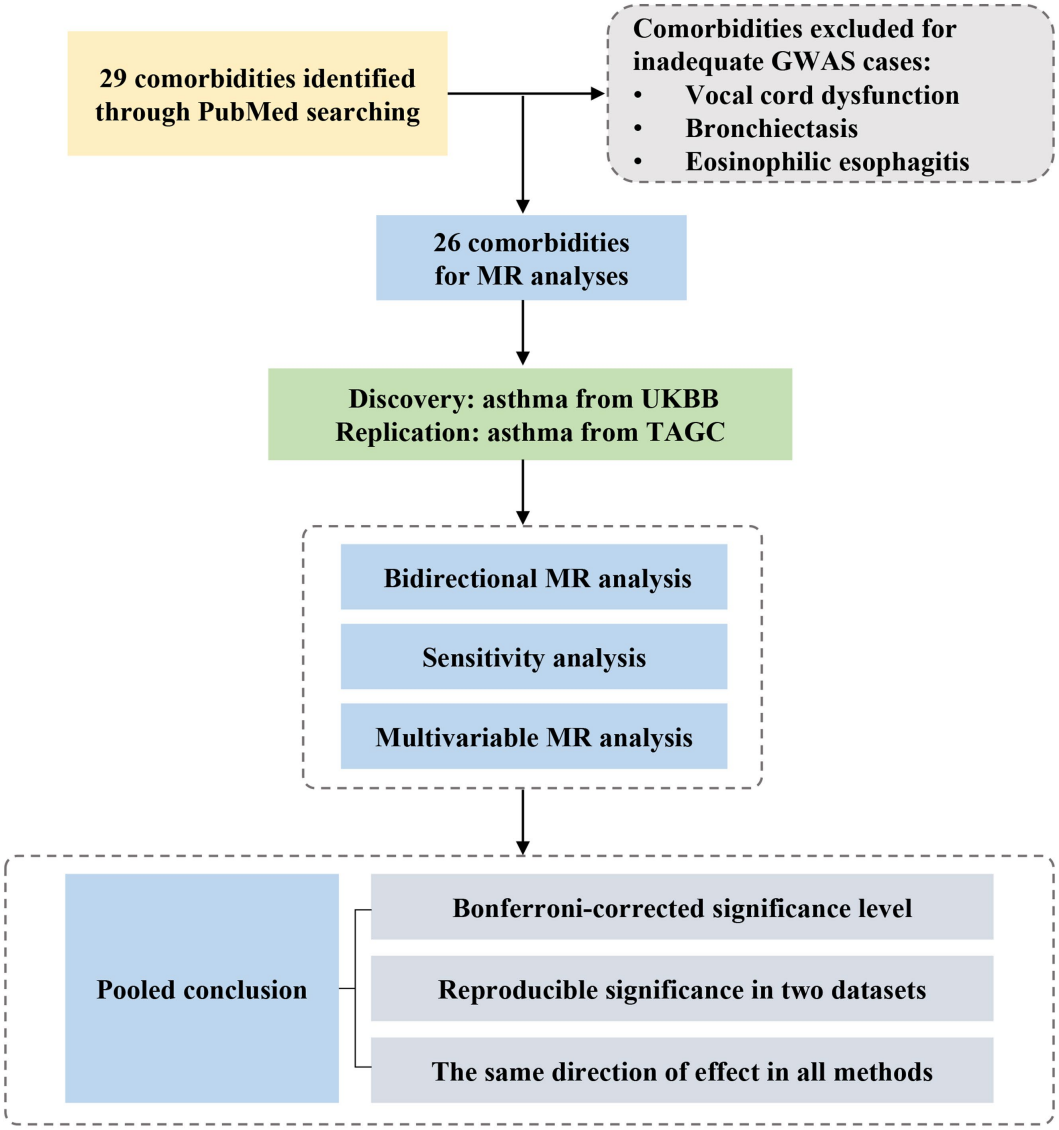
Study design and workflow. GWAS, genome-wide association study; MR, Mendelian randomization; UKBB, UK Biobank; TAGC, Trans-National Asthma Genetic Consortium.

Figure 1 provides an overview of the study design. Initially, we searched Pubmed to identify possible comorbidities with asthma. Then, we explored the associations of comorbidities with asthma in two independent GWAS studies using bidirectional MR analysis. We also validated the results by multivariate Mendelian randomization analysis and several sensitivity analyses. Finally, we combined the discovery and replication datasets and concluded. The analysis process complies with STROBE-MR guidelines (Supplementary Table S1) (16).

### 2.2 Selection of comorbidities

We searched primary studies, meta-analyses, and reviews reporting asthma-related comorbidities from PubMed. All included studies were published in English up to March 1, 2023. The following search terms were used in combination: “asthma,” “comorbidities,” “prevalence,” and “association.” A total of 29 comorbidities were retrieved. After searching the GWAS catalog (17), MRC-IEU OpenGWAS project (18), and the FinnGen study (19), three comorbidities were excluded because complete GWAS summary statistics were not available or contained fewer than 2,000 cases (Table 1).

**Table 1:** Characteristics of the GWAS summary data.

Comorbidity	Data source	Sample size	*N* cases	*N* controls	PMID
**Respiratory tract/pulmonary comorbidities**					
Obstructive sleep apnea	FinnGen-R8	341,071	33,423	307,648	
Chronic obstructive pulmonary disease	FinnGen-R8	299,999	16,410	283,589	
Chronic sinusitis	FinnGen-R8	273,192	14,639	258,553	
**Allergic comorbidities**					
Atopic dermatitis	FinnGen-R8	318,873	11,964	306,909	
Allergic conjunctivitis	FinnGen-R8	342,499	18,321	324,178	
Allergic rhinitis	FinnGen-R8	340,880	9,707	331,173	
Allergic urticaria	FinnGen-R8	333,382	2,112	331,270	
**Cardiovascular comorbidities**					
Hypertension	FinnGen-R8	342,439	98,683	243,756	
Hypertensive heart disease	FinnGen-R8	251,171	7,415	243,756	
Heart failure	FinnGen-R8	341,561	23,622	317,939	
Coronary heart disease	FinnGen-R8	342,499	39,036	303,463	
**Metabolic comorbidities/trait**					
Type 2 diabetes	FinnGen-R8	332,321	49,114	283,207	
Hyperthyroidism	FinnGen-R8	340,964	7,209	333,755	
Autoimmune-related hypothyroidism	FinnGen-R8	287,247	36,321	250,926	
Obesity	FinnGen-R8	342,400	18,330	324,070	
Body mass index	Locke et al. (20) GIANT Consortium	322,154			25673413
**Gastrointestinal comorbidities**					
Gastroesophageal reflux disease	FinnGen-R8	315,123	22,867	292,256	
Peptic ulcer	FinnGen-R8	300,496	8,240	292,256	
Irritable bowel syndrome	FinnGen-R8	284,799	8,116	276,683	
Ulcerative colitis	Liu et al. (21) IIBDGC	27,432	6,968	20,464	26192919
Crohn’s disease	Liu et al. (21) IIBDGC	20,883	5,956	14,927	26192919
**Psychiatric and neurological comorbidities**					
Depression	FinnGen-R8	338,111	38,225	299,886	
Anxiety	FinnGen-R8	329,077	21,519	307,558	
Epilepsy	FinnGen-R8	275,016	10,354	264,662	
Insomnia	FinnGen-R8	340,820	3,514	337,306	
**Musculoskeletal comorbidities**					
Osteoporosis	FinnGen-R8	332,020	6,303	325,717	

GIANT, Genetic Investigation of Anthropometric Traits (GIANT) Consortium; IIBDGC, International Inflammatory Bowel Disease Genetics Consortium.

### 2.3 Data source

At the discovery stage, GWAS summary statistics for asthma were extracted from UK Biobank (including 56,167 asthma cases and 352,255 controls) (22). Asthma cases were defined based on self-reported questionnaires, hospital records (ICD-9 and ICD-10), and primary care records. We also extracted data from the Trans-National Asthma Genetic Consortium (TAGC) for replication, including 56 studies of subjects of European ancestry (19,954 asthma cases and 107,715 controls) (23). Asthma was defined based on doctor’s diagnosis and/or standardized questionnaires.

In two-sample MR, the presence of sample overlap can result in an inflated type 1 error. To avoid sample overlap, instrumental variables of body mass index were obtained from the Genetic Investigation of Anthropometric Traits (GIANT) Consortium (20), inflammatory bowel disease (including ulcerative colitis and Crohn’s disease) data from the International Inflammatory Bowel Disease Genetics Consortium (IIBDGC) (21), and other comorbidities data from the FinnGen study (19). Participants in all studies were of European ancestry.

### 2.4 Selection of instrumental variables

The three main assumptions of Mendelian randomization should be met when selecting instrumental variables (Supplementary Figure S1). We extracted single nucleotide polymorphisms (SNPs) related to each trait at the significance level of *p* < 5 × 10^−8^. Since few SNPs were related to asthma at the *p* < 5 × 10^−8^ level for allergic urticaria, heart failure, gastroesophageal reflux disease, peptic ulcer, irritable bowel syndrome, anxiety, epilepsy, insomnia, and osteoporosis, the genetic instrumentation was set at *p* < 1 × 10^−5^ to perform sensitivity analysis.

To minimize the influence of linkage disequilibrium (LD) between the SNPs, a stringent condition (*r*^2^ < 0.001 and 10,000 kb clumping distance) was set to ensure that the genetic instruments selected were conditionally independent of each other, only the SNPs with the lowest *p*-values were kept. For each SNP, the *F* statistic (*F* = beta^2^/se^2^) was used to assess weak instrumental variable bias.

Next, we extracted exposure IVs from the outcome data and performed data harmonization to ensure that SNPs effects corresponded to the same alleles for both exposure and outcome data. Detailed information on the SNPs used as instrumental variables can be found in Supplementary Tables S2, S3.

### 2.5 Statistical analysis

In this study, the main MR analysis was performed using the multiplicative random-effects inverse-variance weighted (IVW) method, despite the assumption that all SNPs are valid instrumental variables susceptible to potential pleiotropic effects (24). We also repeated the analysis using weighted median (WM) and MR-Egger regression. The WM is based on the assumption that 50% of the information from the IVs is valid (25). MR Egger regression assumes all instrumental variables to be invalid and can produce estimates after accounting for horizontal pleiotropy (26). Then, we assessed the heterogeneity and pleiotropy of individual SNPs by Cochran *Q* statistic and MR-Egger intercept test. We performed a series of sensitivity analyses that allowed for valid estimates when horizontal pleiotropy existed (18). The MR-PRESSO method identifies and corrects outliers and provides adjusted IVW results (27). We also applied Causal Analysis Using Summary Effect Estimates (CAUSE), a new MR method that accounts for correlated and uncorrelated horizontal pleiotropic effects and can avoid more false positives (28). Additionally, given the potential interaction between chronic sinusitis, atopic dermatitis, allergic conjunctivitis, and allergic rhinitis (8, 29), we performed multivariate MR to adjust for potential interactions between these diseases and to estimate their relationship with asthma.

The Bonferroni-corrected significance level of *p* < 1.9 × 10^−3^ (0.05/26 traits) was used. The significant association was defined as the *p*-value less than 1.9 × 10^−3^ in at least one method, with the same direction of effect in the other methods, and which can be repeated in another dataset. *p*-values between 1.9 × 10^−3^ and 0.05 in at least one method were considered to be potential associations, with the same direction of effect in the other methods, and replicable in another data set. Other situations are considered to have no clear association. All statistical analyses were performed using the “TwoSampleMR” (18), “MVMR” (30), and “CAUSE” (28) packages in R Software 4.1.3.

## 3 Results

### 3.1 Overview

**Figure 2:**
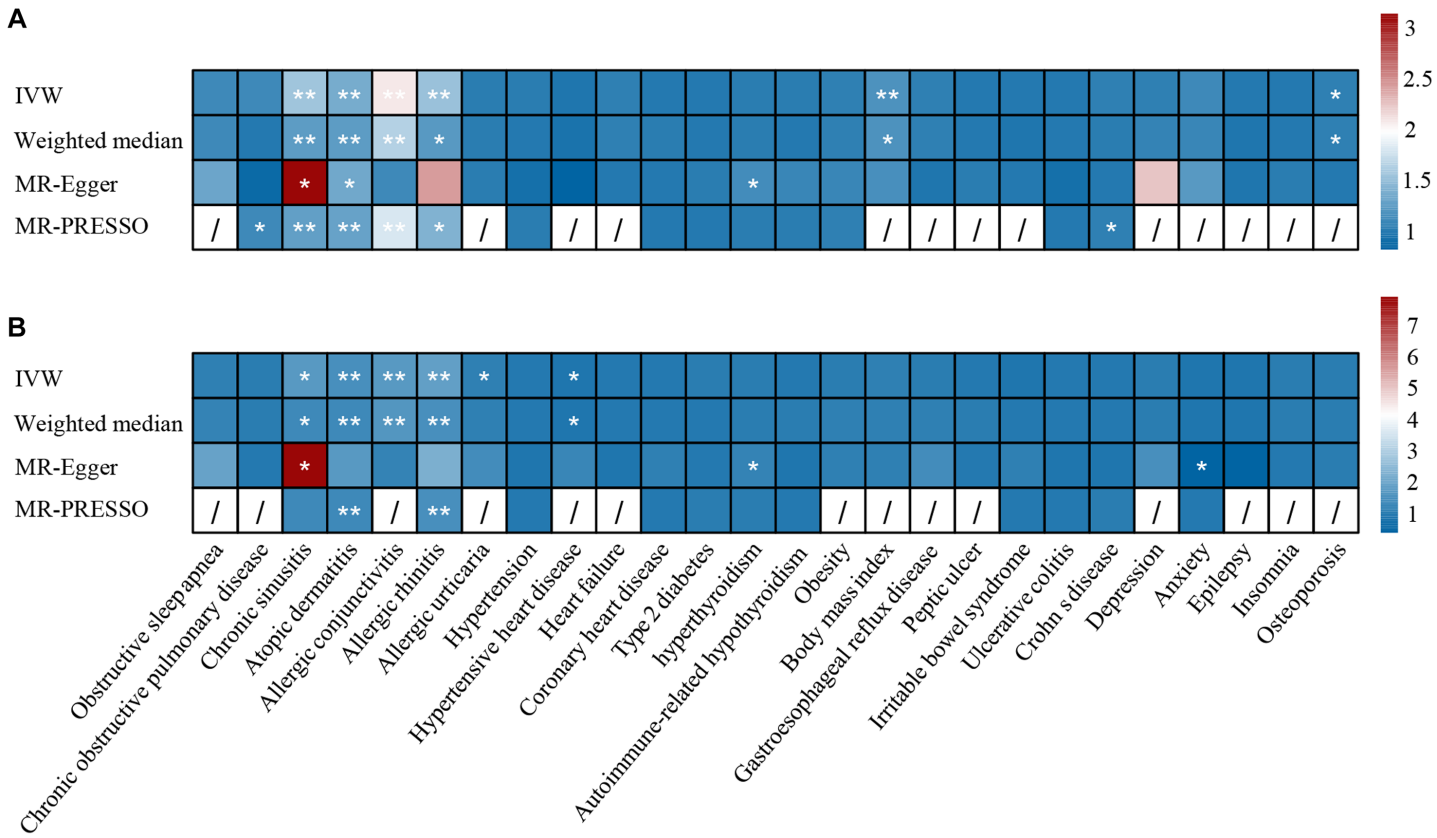
The causal effect of comorbidities on asthma in **(A)** UK Biobank datasets and **(B)** TAGC datasets. *: *p* <0.05, **: *p* < 1.9 × 10^−3^. IVW, inverse-variance weighted; MR-PRESSO, MR pleiotropy residual sum and outlier.

**Figure 3:**
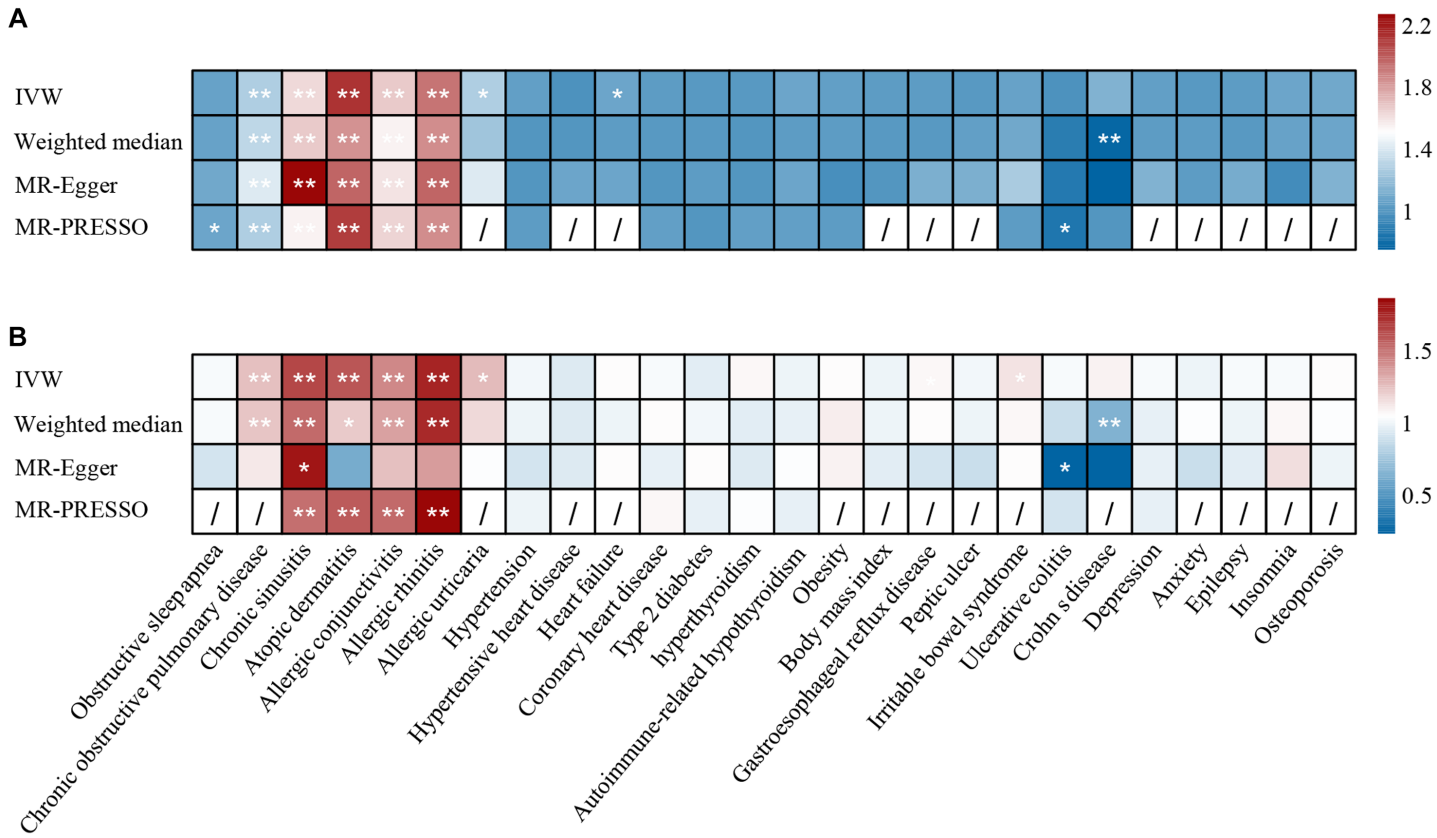
The causal effect of asthma on comorbidities in **(A)** UK Biobank datasets and **(B)** TAGC datasets. *: *p* <0.05, **: *p* < 1.9 × 10^−3^. IVW, inverse-variance weighted; MR-PRESSO, MR pleiotropy residual sum and outlier.

We included 26 comorbidities to explore their potential causal relationship with asthma. The number of SNPs varied from 3 to 163. The obesity trait included the obesity cohort from the FinnGen study and the BMI data from the GIANT consortium. The data sources are summarized in Table 1. The *F*-statistic for each IV was greater than 10, indicating a low likelihood of weak instrument bias (Supplementary Tables S2, S3). The results of the causal association found between comorbidity and asthma in UK Biobank are summarized in Figures 2, 3 and Supplementary Tables S4, S5.

### 3.2 The causal effect of comorbidities on asthma

Regarding the 26 tested comorbidities, genetically predicted chronic sinusitis (UKBB: OR = 1.54, *p* = 1.40 × 10^−5^; TAGC: OR = 1.71, *p* = 7.11 × 10^−3^), atopic dermatitis (UKBB: OR = 1.36, *p* = 9.37 × 10^−21^; TAGC: OR = 1.50, *p* = 9.25 × 10^−11^), allergic conjunctivitis (UKBB: OR = 2.07, *p* = 4.32 × 10^−6^; TAGC: OR = 1.69, *p* = 1.10 × 10^−7^), and allergic rhinitis (UKBB: OR = 1.53, *p* = 5.20 × 10^−6^; TAGC: OR = 1.84, *p* = 1.06 × 10^−5^) were significantly associated with a higher risk of asthma. There was a potential association between hyperthyroidism and the increased risk of asthma (UKBB: OR_MR-Egger_ = 1.12, *p* = 0.04; TAGC: OR_MR-Egger_ = 1.19, *p* = 0.01). Because horizontal pleiotropy was observed in the associations of chronic sinusitis and asthma, we performed MR-PRESSO and CAUSE analysis to evaluate the results. After removing outliers by MR-PRESSO, the relationship remained stable in UKBB (OR = 1.26, *p* = 7.46 × 10^−5^). However, the relationship did not withstand MR-PRESSO corrected in TAGC (OR = 1.32, *p* = 0.094). In the CAUSE-corrected results, this relationship remains robust (UKBB: OR = 1.20, *p* = 1.59 × 10^−6^; TAGC: OR = 1.22, *p* = 0.005).

### 3.3 The causal effect of asthma on comorbidities

We found that genetically predicted asthma had an increased risk of COPD (UKBB: OR = 1.24, *p* = 2.25 × 10^−9^; TAGC: OR = 1.25, *p* = 9.08 × 10^−11^), chronic sinusitis (UKBB: OR = 1.61, *p* = 5.25 × 10^−21^; TAGC: OR = 1.64, *p* = 1.22 × 10^−13^), atopic dermatitis (UKBB: OR = 2.11, *p* = 1.24 × 10^−24^; TAGC: OR = 1.60, *p* = 8.06 × 10^−5^), allergic conjunctivitis (UKBB: OR = 1.65, *p* = 6.66 × 10^−35^; TAGC: OR = 1.44, *p* = 1.50 × 10^−11^), and allergic rhinitis (UKBB: OR = 1.90, *p* = 2.38 × 10^−57^; TAGC: OR = 1.76, *p* = 2.31 × 10^−25^). We also observed possible pleiotropy for the relationship between asthma and chronic sinusitis. The relationship remained stable in MR-PRESSO corrected results (UKBB: OR = 1.53, *p* = 3.73 × 10^−15^; TAGC: OR = 1.51, *p* = 1.13 × 10^−6^). The results were confirmed in CAUSE (UKBB: OR = 1.38, *p* = 1.15 × 10^−12^; TAGC: OR = 1.17, *p* = 0.006). There was potential evidence to support that genetically predicted asthma could increase allergic urticaria risk (UKBB: OR = 1.25, *p* = 0.003; TAGC: OR = 1.27, *p* = 0.017).

### 3.4 Results for multivariable Mendelian randomization

**Figure 4:**
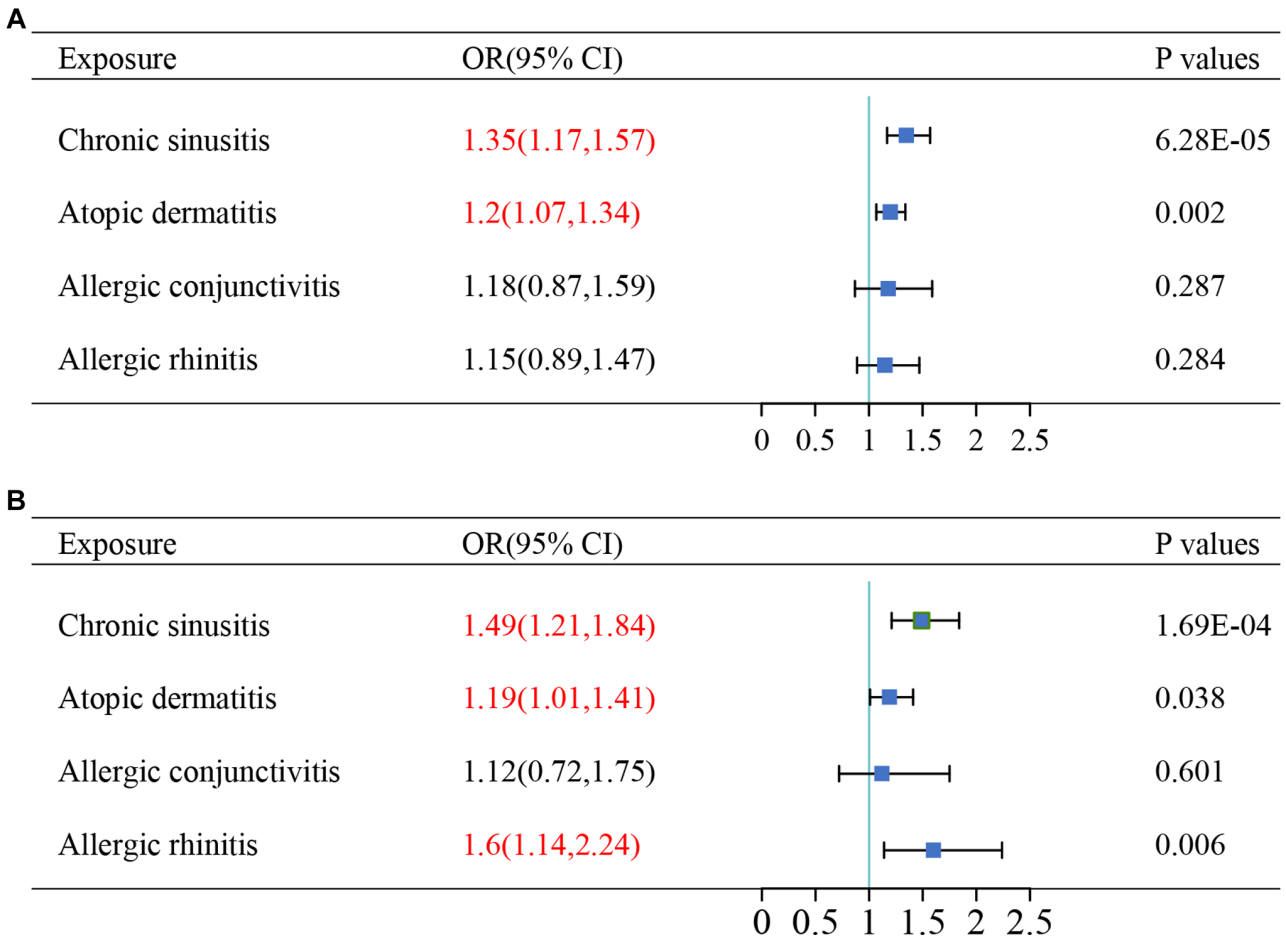
The results of multivariable Mendelian randomizations in **(A)** UK Biobank datasets and **(B)** TAGC datasets. Red text denotes the *P*-value for this comorbidity is less than 0.05. OR, odds ratio.

Since certain phenotypes of chronic sinusitis, atopic dermatitis, allergic conjunctivitis, and allergic rhinitis are characterized by Th2-dominant inflammatory pathways and the coexistence of these diseases has been reported in observational studies (8, 29, 31, 32), we used multivariate analysis to adjust for potential interactions of these diseases. After adjusting for other diseases, chronic sinusitis (UKBB: OR = 1.35, *p* = 6.28 × 10–5; TAGC: OR = 1.49, *p* = 1.69 × 10^−4^) and atopic dermatitis (UKBB: OR = 1.20, *p* = 0.002; TAGC: OR = 1.19, *p* = 0.038) still had an effect on increased asthma risk, and the results were consistent across the two asthma GWAS datasets, suggesting that this causal relationship is robust. In the TAGC dataset, allergic rhinitis was associated with an increased risk of asthma (OR = 1.6, *p* = 0.006), which was not present in the UKBB dataset. For allergic conjunctivitis, there was no significance in either dataset (Figure 4).

## 4 Discussion

In this study, the largest number of comorbidities with asthma was assessed in a Mendelian randomization analysis. There is a bidirectional causal relationship between chronic sinusitis, atopic dermatitis, allergic conjunctivitis, allergic rhinitis and asthma. Hyperthyroidism potentially increases the risk of asthma. In the reverse direction, asthma was significantly associated with a higher risk of chronic obstructive pulmonary disease (COPD) and a potential association with allergic urticaria. Comorbidities make the management and treatment of asthma more difficult. Overall, these findings provide a promising strategy for asthma research and clinical decision-making.

The coexistence of asthma with chronic sinusitis, atopic dermatitis, allergic conjunctivitis, and allergic rhinitis has been reported in previous clinical studies (33, 34). Asthma had a strong and consistent association with chronic sinusitis (35). Abnormal sinus CT scans were found in all patients with severe steroid-dependent asthma and 88% of patients with mild-to-moderate asthma (36). Meanwhile, a cohort study showed that asthma was a major factor in the recurrence of chronic sinusitis (37). 49.7% of patients with atopic dermatitis have combined asthma (38). Up to 40% of patients with allergic rhinitis (AR) report asthma symptoms and up to 80% of patients with asthma have symptomatic AR (39). It has been suggested that type 2 inflammation is a common feature of asthma and many atopic diseases, and similar to asthma, these four diseases are characterized by upregulation of type 2 cytokines, IgE-mediated release of immune mediators, and epithelial or epidermal barrier dysfunction (7, 8). Therapies that target type 2 inflammation, such as dupilumab, have shown improvement in patients with AD, asthma, or CRSwNP (40). These results support the hypothesis that targeting the drivers of type 2 inflammation may be beneficial in a variety of allergic diseases. The unified airway hypothesis proposes that upper and lower airway diseases, such as chronic sinusitis, allergic rhinitis, and asthma, are closely related pathophysiologically, reflecting a single pathologic process manifesting at different locations within the airway (41). However, the relationship between these diseases remains unclear. In this MR study, we found a significant bidirectional causal relationship of asthma with chronic sinusitis, atopic dermatitis, allergic conjunctivitis, and allergic rhinitis. After correction for pleiotropy and multivariate MR, the increased risk of asthma by chronic sinusitis and atopic dermatitis remained significant, providing new directions for the prevention and treatment of asthma and atopic diseases.

Asthma and COPD are the most common lung diseases with a significant economic and health burden. The two diseases share some clinical features and physiological abnormalities, including coughing, wheezing, dyspnea, and airflow obstruction. The asthma-COPD overlap (ACO) has been at the center of the debate for decades, with the Dutch hypothesis suggesting that asthma and COPD are manifestations of the same underlying disease, and the British hypothesis suggesting that the two diseases are distinct entities arising from different mechanisms (42, 43). Some patients with asthma, especially smokers, develop fixed airflow obstruction and COPD in their later years. Some COPD patients have clinical characteristics similar to asthma, but the development of asthma is less common in these patients (44). Similarly, our study did not find a causal relationship between COPD and higher asthma risk, but in the reverse direction, genetic asthma predisposition was significantly associated with increased COPD risk. This finding may provide new ideas for elucidating the pathophysiological mechanisms of ACO.

Previous studies have suggested that thyroid disease may be a non-respiratory comorbidity of asthma (4, 34, 45, 46). Animal studies show reduced allergen-induced inflammatory responses in thyroidectomized rats (47). Thyroid hormone can induce a proliferative airway smooth muscle phenotype that may enhance airway smooth muscle remodeling in asthma (48). In this MR study, we found a potential association between hyperthyroidism and an increased risk of asthma. However, there is a lack of large-scale studies evaluating the relationship between hyperthyroidism and asthma, and further studies are still needed.

A higher incidence of asthma has been found in obese individuals in observational studies (49, 50), with contradictory findings (51–53). In this study, we used the obesity cohort from the FinnGen study and BMI data from the GIANT consortium to investigate the relationship between obesity and asthma. BMI was significantly associated with asthma in the UKBB cohort, but the association was not stable in the TAGC. There was also no evidence of a genetic link between obesity and asthma when using the FinnGen obesity cohort. Another MR study suggested that asthma may be overdiagnosed in obese individuals because of wheezing, which may contribute to previous inconsistent results (54). Overall, a causal relationship between obesity and asthma cannot be determined in our MR study.

Traditional clinical studies are susceptible to confounding factors and various biases. The main advantage of Mendelian randomization is the ability to make causal inferences. Previous Mendelian randomization studies of asthma and comorbidities had some limitations that biased the results of the analysis, such as overlapping samples in the studies that can lead to an increased risk of category 1 error (11, 12), or not validated in another dataset (55–57). Our MR study avoided sample overlap by selecting new large-scale GWAS data, performing several sensitivity analyses, and validating results in another large-scale independent database, which greatly improved the reliability of our conclusions.

Our study also has several limitations. First, as with all MR studies, pleiotropy is a challenge in the MR analysis. As in chronic sinusitis, we conducted several sensitivity analyses and made different assumptions about the possible pleiotropy. Second, we did not perform a correlation analysis between asthma types and severity with comorbidities, as the main objective of this study was to explore the existence of a causal relationship. Finally, given that our findings were restricted to Europeans, the conclusion might not be directly generalized to other populations. To assess the association between comorbidities and asthma in other ethnic groups, further studies are needed.

In conclusion, we have shown that several comorbidities are strongly associated with asthma. Managing these comorbidities through multidisciplinary collaboration may lead to better clinical asthma outcomes. More prospective studies of these comorbidities are needed, which may also have implications for understanding the mechanisms of asthma. There is also a need for clinical trials that investigate the effects of medications targeting the central pathways of allergic disease. Asthma management strategies should be individualized and should not only focus on the asthma itself.

## 5 Conclusion

In this MR study, we comprehensively evaluated the causal relationship between asthma and comorbidities. There is a bidirectional causal relationship between chronic sinusitis, atopic dermatitis, allergic conjunctivitis, allergic rhinitis and asthma. In addition, genetically predicted hyperthyroidism increases the risk of asthma while asthma is associated with an increased risk of COPD and allergic urticaria. This emphasizes the importance of assessing comorbidities as part of routine diagnosis and management, and the need for an integrated, multidisciplinary approach to treatment to address the underlying comorbidities, particularly in allergic disease.

## Data availability statement

The original contributions presented in the study are included in the article/Supplementary material, further inquiries can be directed to the corresponding author.

## Ethics statement

Ethical approval was not required for the study involving humans in accordance with the local legislation and institutional requirements. Written informed consent to participate in this study was not required from the participants or the participants’ legal guardians/next of kin in accordance with the national legislation and the institutional requirements.

## Author contributions

XW was involved in the analysis and interpretation of data and in writing the original draft of the manuscript. YCH was involved in the acquisition of data and the design of the work. YZH and XCL were involved in revising and editing the manuscript. XSL contributed to the design of the study, project administration, and critically edited the manuscript. All authors contributed to the article and approved the submitted version.

## References

[1] Global Initiative for Asthma. Global strategy for asthma management and prevention, (2023). Available at: www.ginasthma.org.

[2] Althoff MD Ghincea A Wood LG Holguin F Sharma S. Asthma and three colinear comorbidities: obesity, osa, and gerd. J Allergy Clin Immunol Pract. (2021) 9:3877–84. doi: 10.1016/j.jaip.2021.09.003, PMID: 34506967 PMC8578370

[3] Stachler RJ. Comorbidities of asthma and the unified airway. Int Forum Allergy Rhinol. (2015) 5:S17–22. doi: 10.1002/alr.21615, PMID: 26335831

[4] Cardet JC Bulkhi AA Lockey RF. Nonrespiratory comorbidities in asthma. J Allergy Clin Immunol Pract. (2021) 9:3887–97. doi: 10.1016/j.jaip.2021.08.027, PMID: 34492402 PMC8631133

[5] Turrin M Rizzo M Bonato M Bazzan E Cosio MG Semenzato U. Differences between early- and late-onset asthma: role of comorbidities in symptom control. J Allergy Clin Immunol Pract. (2022) 10:3196–203. doi: 10.1016/j.jaip.2022.08.007, PMID: 35970446

[6] Hekking PP Amelink M Wener RR Bouvy ML Bel EH. Comorbidities in difficult-to-control asthma. J Allergy Clin Immunol Pract. (2018) 6:108–13. doi: 10.1016/j.jaip.2017.06.008, PMID: 28734857

[7] Gandhi NA Bennett BL Graham NM Pirozzi G Stahl N Yancopoulos GD. Targeting key proximal drivers of type 2 inflammation in disease. Nat Rev Drug Discov. (2016) 15:35–50. doi: 10.1038/nrd4624 26471366

[8] Busse WW Kraft M Rabe KF Deniz Y Rowe PJ Ruddy M. Understanding the key issues in the treatment of uncontrolled persistent asthma with type 2 inflammation. Eur Respir J. (2021) 58:2003393. doi: 10.1183/13993003.03393-2020, PMID: 33542055 PMC8339540

[9] Bardin PG Rangaswamy J Yo SW. Managing comorbid conditions in severe asthma. Med J Aust. (2018) 209:S11–s7. doi: 10.5694/mja18.00196 30453867

[10] Tin A Köttgen A. Mendelian randomization analysis as a tool to gain insights into causes of diseases: a primer. J Am Soc Nephrol. (2021) 32:2400–7. doi: 10.1681/asn.2020121760, PMID: 34135084 PMC8722812

[11] Freuer D Linseisen J Meisinger C. Asthma and the risk of gastrointestinal disorders: a Mendelian randomization study. BMC Med. (2022) 20:82. doi: 10.1186/s12916-022-02283-7, PMID: 35292014 PMC8925069

[12] Ahn K Penn RB Rattan S Panettieri RA Jr Voight BF An SS. Mendelian randomization analysis reveals a complex genetic interplay among atopic dermatitis, asthma, and gastroesophageal reflux disease. Am J Respir Crit Care Med. (2023) 207:130–7. doi: 10.1164/rccm.202205-0951OC, PMID: 36214830 PMC9893317

[13] Sun YQ Brumpton BM Langhammer A Chen Y Kvaløy K Mai XM. Adiposity and asthma in adults: a bidirectional Mendelian randomisation analysis of the Hunt study. Thorax. (2020) 75:202–8. doi: 10.1136/thoraxjnl-2019-213678, PMID: 31611343

[14] Li R Chen Y Zhao A Huang L Long Z Kang W. Exploring genetic association of insomnia with allergic disease and asthma: a bidirectional Mendelian randomization study. Respir Res. (2022) 23:84. doi: 10.1186/s12931-022-02009-6, PMID: 35392909 PMC8991606

[15] Tang P Guo X Chong L Li R. Mendelian randomization study shows a causal effect of asthma on epilepsy risk. Front Immunol. (2023) 14:1071580. doi: 10.3389/fimmu.2023.1071580, PMID: 36860869 PMC9969112

[16] Skrivankova VW Richmond RC Woolf BAR Yarmolinsky J Davies NM Swanson SA. Strengthening the reporting of observational studies in epidemiology using Mendelian randomization: the STROBE-MR statement. JAMA. (2021) 326:1614–21. doi: 10.1001/jama.2021.18236, PMID: 34698778

[17] Buniello A MacArthur JAL Cerezo M Harris LW Hayhurst J Malangone C. The NHGRI-EBI GWAS Catalog of published genome-wide association studies, targeted arrays and summary statistics 2019. Nucleic Acids Res. (2019) 47:D1005–12. doi: 10.1093/nar/gky1120, PMID: 30445434 PMC6323933

[18] Hemani G Zheng J Elsworth B Wade KH Haberland V Baird D. The MR-Base platform supports systematic causal inference across the human phenome. eLife. (2018) 7:7. doi: 10.7554/eLife.34408, PMID: 29846171 PMC5976434

[19] Kurki MI Karjalainen J Palta P Sipilä TP Kristiansson K Donner K. Finngen: unique genetic insights from combining isolated population and national health register data. Nature. (2022) 613:508–18. doi: 10.1038/s41586-022-05473-8 PMC9849126 36653562

[20] The LifeLines Cohort Study, The ADIPOGen Consortium, The AGEN-BMI Working Group, The CARDIOGRAMplusC4D Consortium, The CKDGen Consortium, The GLGC, The ICBP, The MAGIC Investigators, The MuTHER Consortium, The MIGen Consortium, The PAGE Consortium, The ReproGen Consortium, The GENIE Consortium, The International Endogene Consortium Locke AE Kahali B Berndt SI Justice AE Pers TH. Genetic studies of body mass index yield new insights for obesity biology. Nature. (2015) 518:197–206. doi: 10.1038/nature14177, PMID: 25673413 PMC4382211

[21] Liu JZ van Sommeren S Huang H Ng SC Alberts R Takahashi A. Association analyses identify 38 susceptibility loci for inflammatory bowel disease and highlight shared genetic risk across populations. Nat Genet. (2015) 47:979–86. doi: 10.1038/ng.3359, PMID: 26192919 PMC4881818

[22] Valette K Li Z Bon-Baret V Chignon A Bérubé JC Eslami A. Prioritization of candidate causal genes for asthma in susceptibility loci derived from UK Biobank. Commun Biol. (2021) 4:700. doi: 10.1038/s42003-021-02227-6 34103634 PMC8187656

[23] Demenais F Margaritte-Jeannin P Barnes KC Cookson WOC Altmüller J Ang W. Multiancestry association study identifies new asthma risk loci that colocalize with immune-cell enhancer Marks. Nat Genet. (2018) 50:42–53. doi: 10.1038/s41588-017-0014-7, PMID: 29273806 PMC5901974

[24] Burgess S Butterworth A Thompson SG. Mendelian randomization analysis with multiple genetic variants using summarized data. Genet Epidemiol. (2013) 37:658–65. doi: 10.1002/gepi.21758, PMID: 24114802 PMC4377079

[25] Bowden J Davey Smith G Haycock PC Burgess S. Consistent estimation in Mendelian randomization with some invalid instruments using a weighted median estimator. Genet Epidemiol. (2016) 40:304–14. doi: 10.1002/gepi.21965, PMID: 27061298 PMC4849733

[26] Bowden J Davey Smith G Burgess S. Mendelian randomization with invalid instruments: effect estimation and bias detection through Egger regression. Int J Epidemiol. (2015) 44:512–25. doi: 10.1093/ije/dyv080, PMID: 26050253 PMC4469799

[27] Verbanck M Chen CY Neale B Do R. Detection of widespread horizontal pleiotropy in causal relationships inferred from Mendelian randomization between complex traits and diseases. Nat Genet. (2018) 50:693–8. doi: 10.1038/s41588-018-0099-7, PMID: 29686387 PMC6083837

[28] Morrison J Knoblauch N Marcus JH Stephens M He X. Mendelian randomization accounting for correlated and uncorrelated pleiotropic effects using genome-wide summary statistics. Nat Genet. (2020) 52:740–7. doi: 10.1038/s41588-020-0631-4, PMID: 32451458 PMC7343608

[29] McCormick JP Lee JT. Insights into the implications of coexisting type 2 inflammatory diseases. J Inflamm Res. (2021) 14:4259–66. doi: 10.2147/jir.S311640, PMID: 34511966 PMC8416183

[30] Sanderson E Davey Smith G Windmeijer F Bowden J. An examination of multivariable Mendelian randomization in the single-sample and two-sample summary data settings. Int J Epidemiol. (2019) 48:713–27. doi: 10.1093/ije/dyy262, PMID: 30535378 PMC6734942

[31] Khan AH Gouia I Kamat S Johnson R Small M Siddall J. Prevalence and severity distribution of type 2 inflammation-related comorbidities among patients with asthma, chronic rhinosinusitis with nasal polyps, and atopic dermatitis. Lung. (2023) 201:57–63. doi: 10.1007/s00408-023-00603-z, PMID: 36808551 PMC9968259

[32] Thyssen JP Toft PB Halling-Overgaard AS Gislason GH Skov L Egeberg A. Incidence, prevalence, and risk of selected ocular disease in adults with atopic dermatitis. J Am Acad Dermatol. (2017) 77:280–6.e1. doi: 10.1016/j.jaad.2017.03.003, PMID: 28601391

[33] Cohen S Berkman N Picard E Levi T Derazne E Tzur D. Co-morbidities and cognitive status in a cohort of teenagers with asthma. Pediatr Pulmonol. (2016) 51:901–7. doi: 10.1002/ppul.23443, PMID: 27177276

[34] Rogliani P Laitano R Ora J Beasley R Calzetta L. Strength of association between comorbidities and asthma: a meta-analysis. Eur Respir Rev. (2023) 32:220202. doi: 10.1183/16000617.0202-2022 36889783 PMC10032614

[35] Jarvis D Newson R Lotvall J Hastan D Tomassen P Keil T. Asthma in adults and its association with chronic rhinosinusitis: the Ga2len survey in Europe. Allergy. (2012) 67:91–8. doi: 10.1111/j.1398-9995.2011.02709.x 22050239

[36] Bresciani M Paradis L Des Roches A Vernhet H Vachier I Godard P. Rhinosinusitis in severe asthma. J Allergy Clin Immunol. (2001) 107:73–80. doi: 10.1067/mai.2001.111593 11149994

[37] Sella GCP Tamashiro E Sella JA Aragon DC Mendonça TN Arruda LKP. Asthma is the dominant factor for recurrence in chronic rhinosinusitis. J Allergy Clin Immunol Pract. (2020) 8:302–9. doi: 10.1016/j.jaip.2019.08.007, PMID: 31425833

[38] Silverberg JI Gelfand JM Margolis DJ Boguniewicz M Fonacier L Grayson MH. Association of atopic dermatitis with allergic, autoimmune, and cardiovascular comorbidities in us adults. Ann Allergy Asthma Immunol. (2018) 121:604–12.e3. doi: 10.1016/j.anai.2018.07.042, PMID: 30092266 PMC13217624

[39] Leynaert B Neukirch C Kony S Guénégou A Bousquet J Aubier M. Association between asthma and rhinitis according to atopic sensitization in a population-based study. J Allergy Clin Immunol. (2004) 113:86–93. doi: 10.1016/j.jaci.2003.10.010, PMID: 14713912

[40] Canonica GW Bourdin A Peters AT Desrosiers M Bachert C Weidinger S. Dupilumab demonstrates rapid onset of response across three type 2 inflammatory diseases. J Allergy Clin Immunol Pract. (2022) 10:1515–26. doi: 10.1016/j.jaip.2022.02.026, PMID: 35259535

[41] Bachert C Luong AU Gevaert P Mullol J Smith SG Silver J. The unified airway hypothesis: evidence from specific intervention with anti-Il-5 biologic therapy. J Allergy Clin Immunol Pract. (2023) 11:2630–41. doi: 10.1016/j.jaip.2023.05.011 37207831

[42] Soler X Ramsdell JW. Are asthma and COPD a continuum of the same disease? J Allergy Clin Immunol Pract. (2015) 3:489–95; quiz 96-7. doi: 10.1016/j.jaip.2015.05.030 26164572

[43] Milne S Mannino D Sin DD. Asthma-COPD overlap and chronic airflow obstruction: definitions, management, and unanswered questions. J Allergy Clin Immunol Pract. (2020) 8:483–95. doi: 10.1016/j.jaip.2019.10.044, PMID: 31740296

[44] Marcon A Locatelli F Dharmage SC Svanes C Heinrich J Leynaert B. The coexistence of asthma and COPD: risk factors, clinical history and lung function trajectories. Eur Respir J. (2021) 58:2004656. doi: 10.1183/13993003.04656-2020 33863744 PMC8613837

[45] Weare-Regales N Chiarella SE Cardet JC Prakash YS Lockey RF. Hormonal effects on asthma, rhinitis, and eczema. J Allergy Clin Immunol Pract. (2022) 10:2066–73. doi: 10.1016/j.jaip.2022.04.002 35436605 PMC9392967

[46] Settipane GA Schoenfeld E Hamolsky MW. Asthma and hyperthyroidism. J Allergy Clin Immunol. (1972) 49:348–55. doi: 10.1016/0091-6749(72)90133-9, PMID: 4554357

[47] Manzolli S Macedo-Soares MF Vianna EO Sannomiya P. Allergic airway inflammation in hypothyroid rats. J Allergy Clin Immunol. (1999) 104:595–600. doi: 10.1016/s0091-6749(99)70329-5 10482833

[48] Dekkers BG Naeimi S Bos IS Menzen MH Halayko AJ Hashjin GS. L-thyroxine promotes a proliferative airway smooth muscle phenotype in the presence of TGF-Β1. Am J Physiol Lung Cell Mol Physiol. (2015) 308:L301–6. doi: 10.1152/ajplung.00071.2014, PMID: 25480330

[49] Wickens K Barry D Friezema A Rhodius R Bone N Purdie G. Obesity and asthma in 11–12 years old New Zealand children in 1989 and 2000. Thorax. (2005) 60:7–12. doi: 10.1136/thx.2002.001529, PMID: 15618575 PMC1747164

[50] von Mutius E Schwartz J Neas LM Dockery D Weiss ST. Relation of body mass index to asthma and atopy in children: the National Health and Nutrition Examination Study III. Thorax. (2001) 56:835–8. doi: 10.1136/thorax.56.11.835, PMID: 11641506 PMC1745951

[51] Barranco P García-Río F Olivares J López-Carrasco V Alvarez-Sala R Quirce S. Asthma diagnosis is not associated with obesity in a population of adults from Madrid. J Investig Allergol Clin Immunol. (2011) 21:540–5. PMID: 22312938

[52] Stanley AH Demissie K Rhoads GG. Asthma development with obesity exposure: observations from the cohort of the National Health and Nutrition Evaluation Survey Epidemiologic Follow-up Study (NHEFS). J Asthma. (2005) 42:97–9. doi: 10.1081/jas-51338 15871440

[53] Schachter LM Salome CM Peat JK Woolcock AJ. Obesity is a risk for asthma and wheeze but not airway hyperresponsiveness. Thorax. (2001) 56:4–8. doi: 10.1136/thorax.56.1.4, PMID: 11120896 PMC1745919

[54] Çolak Y Afzal S Lange P Nordestgaard BG. Obese individuals experience wheezing without asthma but not asthma without wheezing: a Mendelian randomisation study of 85,437 adults from the Copenhagen general population study. Thorax. (2016) 71:247–54. doi: 10.1136/thoraxjnl-2015-207379 26504195

[55] Li Y Wang W Zhou D Lu Q Li L Zhang B. Mendelian randomization study shows a causal effect of asthma on chronic obstructive pulmonary disease risk. PLoS One. (2023) 18:e0291102. doi: 10.1371/journal.pone.0291102, PMID: 37656706 PMC10473539

[56] Tang Z Shen M Xiao Y Liu H Chen X. Association between atopic dermatitis, asthma, and serum lipids: a UK Biobank based observational study and Mendelian randomization analysis. Front Med. (2022) 9:810092. doi: 10.3389/fmed.2022.810092 PMC8899503 35265637

[57] Xu S Gilliland FD Conti DV. Elucidation of causal direction between asthma and obesity: a bi-directional Mendelian randomization study. Int J Epidemiol. (2019) 48:899–907. doi: 10.1093/ije/dyz070, PMID: 31005996 PMC6659368

