# Supplementary figures and images for: The associations between asthma and common comorbidities: a comprehensive Mendelian randomization study
Source: Front Med (Lausanne). 2023 Nov 15;10:1251827. doi: 10.3389/fmed.2023.1251827 (PMC10684927; doi:10.3389/fmed.2023.1251827)

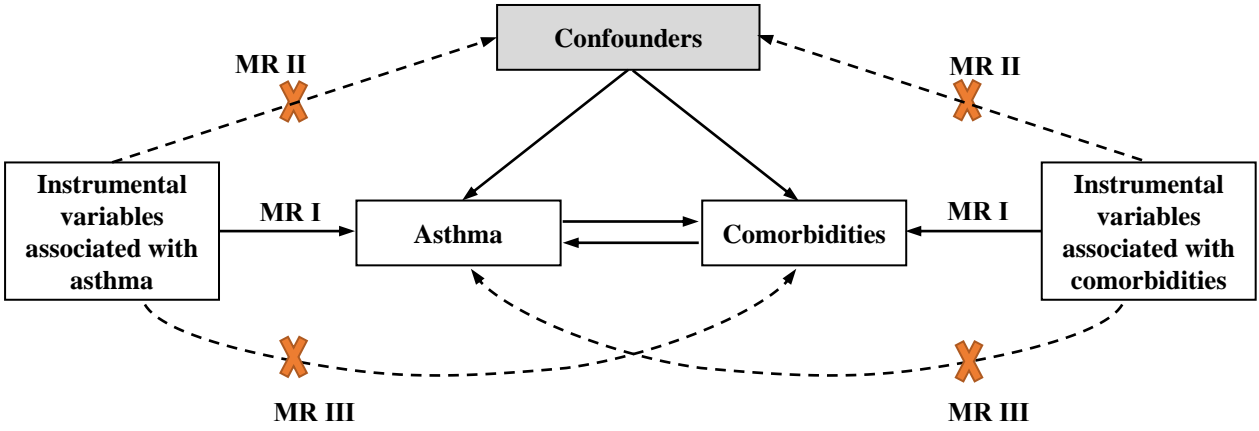

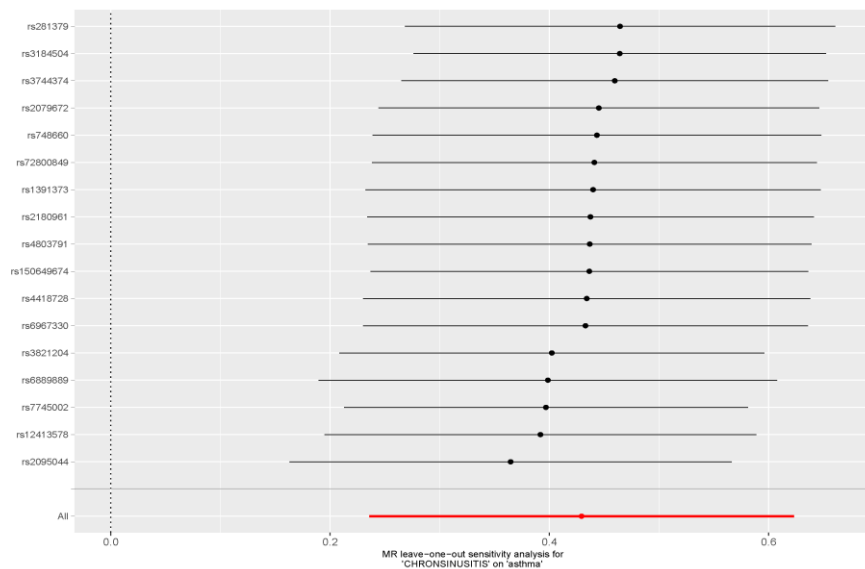

Supplement: Supplementary file 5 [file Data_Sheet_1.PDF]
